# Genotypes Predispose Phenotypes—Clinical Features and Genetic Spectrum of *ABCA4*-Associated Retinal Dystrophies

**DOI:** 10.3390/genes11121421

**Published:** 2020-11-27

**Authors:** Yu-Chi Sung, Chang-Hao Yang, Chung-May Yang, Chao-Wen Lin, Ding-Siang Huang, Yu-Shu Huang, Fung-Rong Hu, Pei-Lung Chen, Ta-Ching Chen

**Affiliations:** 1 Department of Medical Education, National Taiwan University Hospital, Taipei 100, Taiwan; kitty51631@gmail.com; 2 Department of Ophthalmology, National Taiwan University Hospital, Taipei 100, Taiwan; chyangoph@ntu.edu.tw (C.-H.Y.); chungmay@ntu.edu.tw (C.-M.Y.); wesley.lin1@gmail.com (C.-W.L.); sean.maxen@gmail.com (D.-S.H.); amy10191911@gmail.com (Y.-S.H.); fungronghu@ntu.edu.tw (F.-R.H.); 3 Department of Ophthalmology, College of Medicine, National Taiwan University, Taipei 100, Taiwan; 4 Graduate Institute of Clinical Medicine, College of Medicine, National Taiwan University, Taipei 100, Taiwan; 5 Graduate Institute of Medical Genomics and Proteomics, College of Medicine, National Taiwan University, Taipei 100, Taiwan; 6 Department of Medical Genetics, National Taiwan University Hospital, Taipei 100, Taiwan

**Keywords:** *ABCA4*, Stargardt disease 1, retinitis pigmentosa, inherited retinal degeneration, genotype–phenotype correlation

## Abstract

The *ABCA4* gene is one of the most common disease-causing genes of inherited retinal degeneration. In this study, we report different phenotypes of *ABCA4*-associated retinal dystrophies in the Taiwanese population, its clinical progression, and its relationship with genetic characteristics. Thirty-seven subjects were recruited and all patients underwent serial ophthalmic examinations at a single medical center. Fundus autofluorescence (FAF) images were quantified for clinical evaluation, and panel-based next-generation sequencing testing was performed for genetic diagnosis. Visual preservation, disease progression, and genotype–phenotype correlation were analyzed. In this cohort, *ABCA4*-associated retinal degeneration presented as Stargardt disease 1 (STGD1, 62.16%), retinitis pigmentosa (32.43%), and cone-rod dystrophy (5.41%). STGD1 could be further divided into central and dispersed types. In each phenotype, the lesion areas quantified by FAF increased with age (*p* < 0.01) and correlated with poorer visual acuity. However, three patients had the foveal sparing phenotype and had relatively preserved visual acuity. Forty-two *ABCA4* variants were identified as disease-causing, with c.1804C>T (p.Arg602Trp) the most frequent (37.84%). Patients with a combination of severe/null variants could have more extensive phenotypes, such as arRP and dispersed STGD1. This is the first cohort study of *ABCA4*-associated retinal degeneration in Taiwan with wide spectrums of both genotypic and phenotypic characteristics. An extremely high prevalence of c.1804C>T, which has not been reported in East Asia before, was noted. The extensiveness of retinal involvement might be regarded as a spectrum of *ABCA4*-associated retinal dystrophies. Different types of genetic variations could lead to distinctive phenotypes, according to the coding impact of variants.

## 1. Introduction

*ABCA4*, belonging to the ABCA subfamily of ATP-binding cassette transporters (ABC transporters), is located on chromosome 1p22.1, containing 50 exons and encoding 2273 amino acid proteins [1,2]. Similar to other ABC transporters, *ABCA4* functions as an exporter to translocate *N*-retinylidene-phosphatidylethanolamine and phosphatidylethanolamine from the lumen to the cytoplasmic side of photoreceptor disc membranes [3]. Once its function is impaired, the accumulation of toxic retinoid compounds cause retinal pigment epithelium (RPE) and photoreceptor dysfunction [2].

*ABCA4* is one of the most common disease-causing genes in inherited retinal degeneration (IRD) [4], with a prevalence of approximately 1 in 10,000 people, and is considered the main cause of Stargardt disease 1 (STGD1; OMIM 248200) [5,6]. In addition, *ABCA4* mutations are also responsible for retinitis pigmentosa (RP) [7], cone-rod dystrophy (CRD) [8], and a risk factor of age-related macular degeneration (AMD) [9,10]. Patients with *ABCA4*-associated retinal degeneration commonly present with progressive bilateral central vision loss. Symptoms more commonly develop in the second or third decade of life. The severity of disease-causing variants in *ABCA4* is reported to be associated with the onset age of *ABCA4*-associated retinal degeneration. For example, deleterious *ABCA4* variants lead to early-onset disease and poor visual acuity (VA) [6,11].

More than 1200 different variants of *ABCA4*, including missense, splicing, truncating, and frameshift alterations, have been reported in the Leiden Open Variation Database (https://databases.lovd.nl/shared/genes/ABCA4). Disease severity is believed to be associated with residual *ABCA4* functions [12], and different genotypic compositions may lead to distinguished phenotypes of inherited retinal diseases [13,14]. A relationship between disease severity and the ABCA4 genotype revealed that deleterious ABCA4 mutations damage photoreceptors and RPE, known as RP, and the mildest genotype develops into AMD [8,15]. It is important to know more about the genotype–phenotype correlation and the progressive pattern for patients with *ABCA4*-associated retinal degeneration.

Taiwan is an isolated island in East Asia, with a population of approximately 23 million people [16]. The majority (>95%) of Taiwanese are of Han Chinese ancestry that emigrated from continental East Asia, whereas about 2% are of aboriginal ancestry, Austronesians [17]. Therefore, the population in Taiwan is geographically isolated and relatively homogeneous in terms of genetics. Few studies regarding *ABCA4*-associated retinal degeneration have been conducted in the Taiwanese population. In this study, we recruited patients with *ABCA4*-associated retinal dystrophies from the Taiwan IRD project (TIP), which included all patients with IRD with a clinical evaluation and genetic diagnosis via capture-based next-generation sequencing (NGS) testing. We aimed to explore the clinical features, genetic spectrum, and genotype–phenotype correlations of *ABCA4*-associated retinal dystrophies in Taiwan.

## 2. Materials and Methods

### 2.1. Subjects and Clinical Evaluation

The patients included in the present study were recruited as part of the TIP project, which was approved by the Research Ethics Committee of the National Taiwan University Hospital (IRB No.: 201408082RINC). From July 2015 to June 2020, 501 families identified as having IRD were recruited into the TIP project. Among the patients, those who met the inclusion criteria, including (1) harboring two alleles of *ABCA4* disease-causing variants, (2) clinically diagnosed with IRD, and (3) having complete and serial fundus imaging, were recruited.

In total, 37 subjects from 31 unrelated families were enrolled in the present study. All patients recruited were of Han Chinese origin, according to self-reports. Subjects recruited in our program had a series of comprehensive ophthalmic examinations in the Department of Ophthalmology, National Taiwan University Hospital, including best-corrected VA measurement, electroretinograms, color fundus photography, optical coherence tomography, and fundus autofluorescence imaging (FAF). All recruited patients must have had FAF examinations at least twice, with an interval equal or more than 6 months in order to observe the progression of disease. The definite diagnosis of each subject was established according to the above examinations and clinical presentations. We selected the right eye of each patient for statistical analysis. Genomic deoxyribonucleic acid (DNA) was extracted from peripheral blood leukocytes and then sequenced by panel-based NGS for genetic diagnosis.

### 2.2. Next-Generation Sequencing

The blood samples of patients were collected after obtaining informed consent. Genomic DNA was extracted from the peripheral blood leukocytes using a DNA extraction kit (Gentra Puregene Blood Kit, QIAGEN, Hilden, Mettmann, Germany). Genetic testing was performed via a probe capture-based NGS approach targeting 212 IRD-associated genes. The 212 genes were selected from the RetNet database (https://sph.uth.edu/retnet/), OMIM database (https://www.ncbi.nlm.nih.gov/omim), and publications (PubMed search queries: hereditary retinal dystrophy). Targeted enrichment using probe capture was performed to target all transcripts of the 212 genes (Supplementary Table S1). In addition to the capture of exons, we captured the entire genomic sequence, including both exons and introns in some genes (*USH2A*, *OFD1*, *ABCA4*, *PRPF31 CEP290*, *RPGR*, *GUCY2D*, *KCNV2*, *CNGB3*, *CNGA3*, *PRPF4*, *MYO7A*, *RPGRIP1*, *RDH12*, *AIPL1*, *CNGB1*, *NRL*, *SPATA7*, *FAM161A*, *RPE65*, *PDE6A*, *PDE6B*, *BBS10*, *BBS1*). Paired-end sequencing was used for sequencing the captured DNA with an Illumina MiSeq or NextSeq system where appropriate (Illumina Inc., San Diego, CA, USA), and the reads were analyzed for mapping to the human genome reference (February 2009 GRCh37/hg19). Then, variant calling and annotation were performed in silico. Variant filtering was performed by filtering out variants with allele frequencies of more than 5% in either one of the population databases. The pathogenicity of retained variants was predicted using pathogenicity prediction algorithms, including SIFT, PolyPhen-2, MutationTaster, and PROVEAN, and characterized by the American College of Medical Genetics and Genomics guidelines [18]. We used the TAIGenomics platform (https://taigenomics.tw/) and proprietary scripts to conduct and stream different steps of the bioinformatics pipeline. Sanger sequencing was used to confirm the nucleotide change of variants that met the criteria listed above. ABCA4 was the strongest disease-causing gene among our 212 gene panel for every patient recruited in this cohort.

### 2.3. Classification System

**Figure 1:**
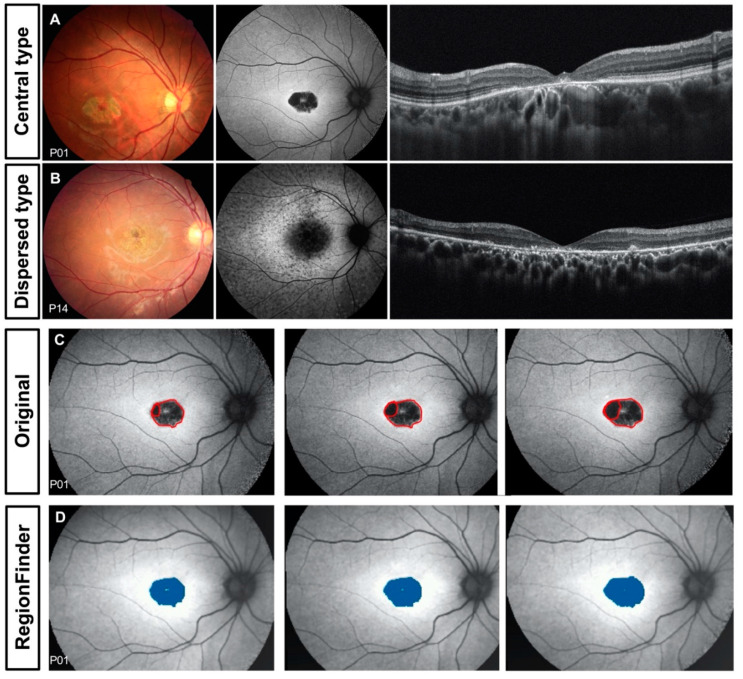
Classification and quantification of fundus autofluorescence. (**A**) A sample case of central type Stargardt disease 1 with a lesion restricted in the central macula. Decreased autofluorescence and retinal pigment epithelium atrophy in the central macula area can be seen. (**B**) A sample case of dispersed Stargardt disease 1 with a diffused hypofluorescence area without a clear margin and heterogeneous background. (**C**) Original series fundus autofluorescence image of patient P01; (**D**) semi-automated recognition by a single grader of the decreased autofluorescence area of P01 using RegionFinder software.

In our report, the 23 patients with STGD1 were subdivided into two groups based on their lesion distribution: central (*n* = 15) and dispersed (*n* = 8; Figure 1). The macular lesion in patients with central type STGD1 was a single bull’s eye lesion, which was restricted to the foveal region with a clear margin, and no obvious fleck was observed (Figure 1A). Patients with dispersed type STGD1 had all other manifestations from macular-confined lesions to those extending outside the macula, with varying degrees of background heterogeneity (Figure 1B).

Genetic characteristics of patients with STGD1 were classified into three types based on the coding impact of *ABCA4* variants [19]. Genotype A was defined as a patient harboring two severe/null variants; genotype B was defined as a patient harboring a severe/null variant and a missense or in-frame insertion/deletion variant; genotype C was defined as a patient who carries two missense or in-frame insertion/deletion variants. Severe/null variants indicate variants that are predicted to affect splicing or lead truncating codons in the ABCA4 protein, such as frameshift, intronic mutations at the splicing site, and nonsense mutations.

### 2.4. Lesion Area Measurement with Fundus Autofluorescence Examination (FAF)

A semi-automated RegionFinder software (Heidelberg Engineering) was utilized to quantify the decreased autofluorescence (DAF) area in the FAF image, according to established protocols [20]. Decreased autofluorescence can simply classify the lesions into two types: definite decreased autofluorescence (DDAF) means lesion area with a level of darkness of almost 100% in reference to the optic nerve, and questionably decreased autofluorescence (QDAF) indicates a lesion with a darkness level of 50–90%, while retinal background serves as the endpoint of scale [21,22]. All of the measurements was done by a single trained grader, and a shadow function in the RegionFinder allowed the operator to brighten the uneven illumination background of the image. In the present study, we took the sum of QDAF and DDAF as the lesion areas for further analysis (Figure 1C,D).

The progression rate of the lesion area was calculated by increment (or decrement) of the involved area compared to the previous visit divided by the interval from the last visit.

### 2.5. Statistical Analysis

The results are shown as the mean ± standard error of the mean, with a 95% confidence interval. A student’s t-test, analysis of variance, and Pearson’s correlation, followed by post hoc multiple comparisons, were performed using SPSS version 27.0 (SPSS Inc., Chicago, IL, USA).

## 3. Results

### 3.1. Demographics

A total of 37 subjects with two alleles of *ABCA4* variants were enrolled. Fifteen were male (40.54%), and 22 were female (59.46%). The clinical phenotypes included Stargardt disease 1 (STGD1, 23/37, 62.16%), CRD, 2/37, 5.41%), and RP (12/37, 32.43%). Their ages ranged from 3 to 77 years old, with a mean age of 34.35 (SD ± 6.82) years. The mean follow-up period was 5.14 (SD ± 2.22) years. The demographic data of the two major phenotypes in this study, STGD1 and RP, are shown in Table 1. There was no statistical significance among the three groups in onset age, but patients of RP sought medical advice later (*p* = 0.038). The average central vision was poorer in patients with RP than in those with STGD1 (*p* < 0.001 at the first visit and *p* = 0.033 at the most recent visit).

### 3.2. Clinical Presentations of ABCA4-Associated Stargardt Disease 1

**Figure 2:**
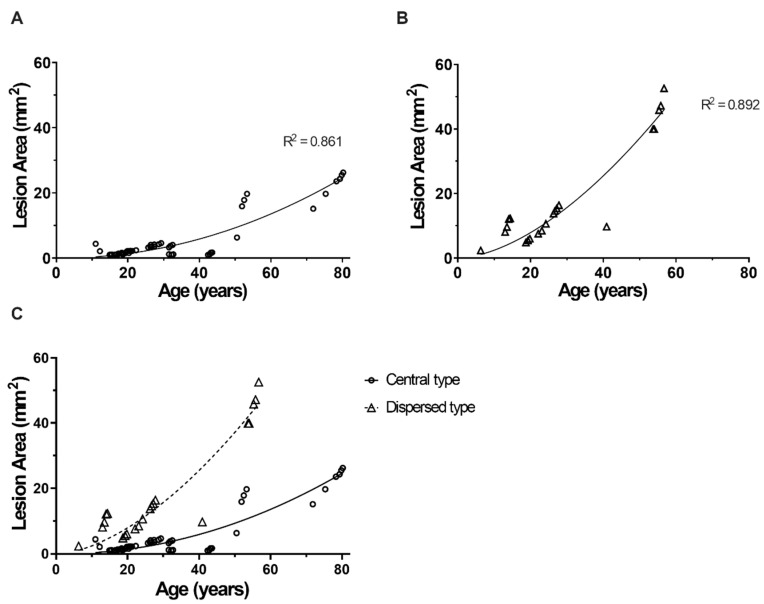
Fundus autofluorescence lesion areas in patients with Stargardt disease 1 across different age groups. (**A**) Central type Stargardt disease 1. (**B**) Dispersed type Stargardt disease 1. (**C**) Comparison of the central type and dispersed type lesion areas in different age groups.

Fourteen central and eight dispersed patients in this cohort received regular FAF imaging during their follow-up period (mean ± SD = 68.05 ± 48.74 months). The involved areas in the central and dispersed types were 5.8 ± 4.35 mm^2^ and 18.67 ± 24.37 mm^2^, respectively, in the most recent autofluorescence imaging. The involved areas of each patient at each visit in both types are plotted in Figure 2A,B. Age was significantly positively correlated with the FAF lesion area (*p* < 0.01* in both groups). This trend seemed to be steeper in the dispersed type (Figure 2C), leading to a larger lesion area in dispersed type Stargardt disease 1.

**Figure 3:**
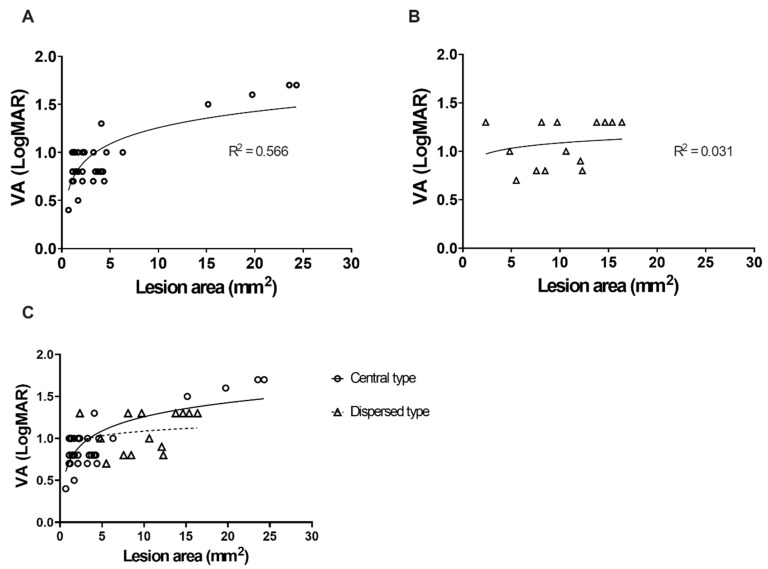
Fundus autofluorescence lesion areas and the visual acuity of patients with Stargardt disease 1. (**A**) Central type Stargardt disease 1. (**B**) Dispersed type Stargardt disease 1. (**C**) Comparison of the central type and dispersed type lesion areas and visual acuity of each patient.

The best-corrected VA at each visit in both types is plotted in Figure 3A,B. During the most recent visit, the mean LogMAR VA was 0.9 ± 0.19 and 0.96 ± 0.65 for the central and dispersed types, respectively (*p* = 0.24). VA seemed to be more susceptible to the increased area involved in the central type (*p* < 0.01*; Figure 3C). Two distinctive cases (P13, P29) with the foveal sparing phenotype of Stargardt disease 1 had larger lesion areas, but preserved VA was excluded in our analysis, and will be discussed independently in the following paragraphs.

**Figure 4:**
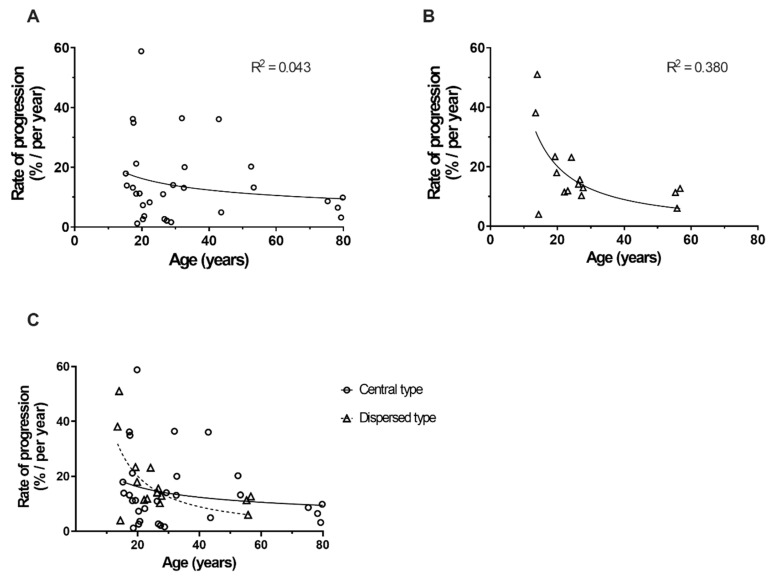
Annual lesion progression rate in patients with Stargardt disease 1 across different age groups. (**A**) Central type Stargardt disease 1. (**B**) Dispersed type Stargardt disease 1. (**C**) Comparison of the central type and dispersed type lesion areas in different age groups. Note: progression rate is calculated using the increment (or decrement) of the previous visit lesion areas in fundus autofluorescence divided by the interval time from the last visit.

Dispersed Stargardt disease 1 was found to have a more rapid progression rate in the involved area. The progression rate of the area involved at each time point of each patient was calculated using the increment (or decrement) percentage of the previous visit divided by the interval from the last visit, which is plotted in Figure 4A,B, respectively. The central and dispersed types’ annual progression rates were 14.19 ± 4.61% and 17.57 ± 6.27%, respectively. Both groups displayed a rapid progression rate during adolescence and a slow progression rate during aging. The dispersed type seemed to have a more significant decline in progression rate than the central type when the patient was older (Figure 4C).

### 3.3. Foveal Sparing Phenotype of Stargardt Disease 1

**Figure 5:**
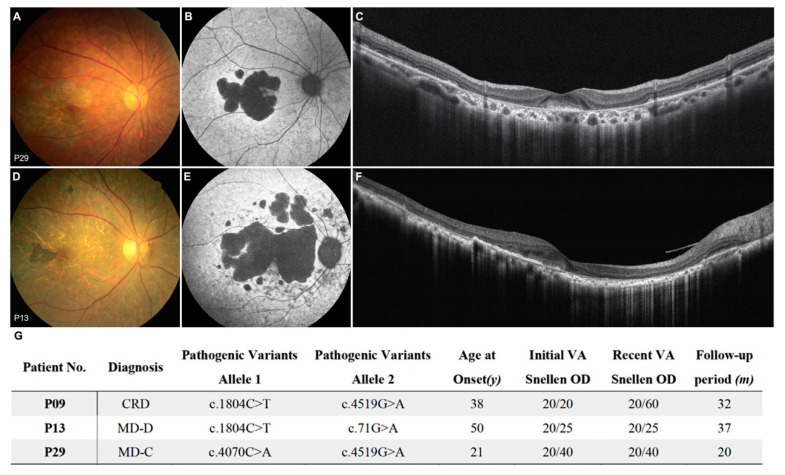
Foveal sparing phenotype. (**A**) P29 with macular chorioretinal atrophy sparing central fovea, (**B**) well demarcated patchy definite decreased autofluorescence (DDAF) with relative hyperautofluorescence in the foveal area, (**C**) loss of the photoreceptor and ellipsoid zone at the parafoveal area with a preserved structure in the central fovea. (**D**) P13 has diffuse chorioretinal atrophy sparing fovea with pigmentation, (**E**) multiple patchy DDAF without foveal involvement and heterogeneous background, (**F**) Diffuse loss of the photoreceptor and ellipsoid zone but a relatively preserved fovea. (**G**) Demographic data of three patients with foveal sparing phenotype in fundus autofluorescence.

**Figure 6:**
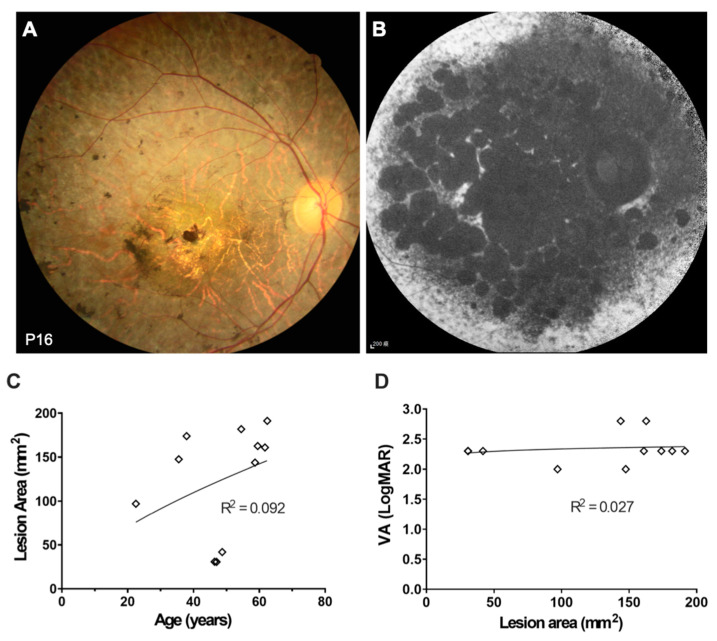
Clinical presentations of *ABCA4*-associated retinitis pigmentosa. (**A**) Fundus color of diffuse chorioretinal atrophy with bone spicule pigmentation and central macula involvement. (**B**) Fundus autofluorescence with diffusely decreased autofluorescence and central macula involvement. (**C**) A comparison of age and the lesion area showed an increment of the lesion area accompanied with aging. (**D**) Poor visual acuity was noted in the retinitis pigmentosa patient group despite the lesion area.

Two of our STGD1 patients displayed relatively better VA compared to the others, despite their FAF examinations revealing larger DDAFs (Figure 5). P13 is grouped in central type, with a LogMAR VA of 0.3 (15th percentile in the central group) and lesion area of 19.71 mm^2^ (57th percentile in the dispersed group). P29 is classified as the dispersed type, with a LogMAR VA of 0.1 (0th percentile in the dispersed group) and lesion area of 52.57 mm^2^ (92nd percentile in the dispersed group). Both had a triangular patch of cell preservation that extended from the paracentral area to the fovea. Despite Stargardt disease 1, one CRD patient (P09) also showed a similar foveal sparing pattern, with preserved VA (initial VA: 20/20 in both eyes; Figure 6D–F). Later disease onset was also noted in the foveal sparing phenotype, and a detailed demographic presentation is shown in Figure 5G.

### 3.4. Clinical Presentations of ABCA4-Associated Retinitis Pigmentosa

Twelve patients in our cohort presented with *ABCA4*-associated RP (Figure 6A,B). They had a similar onset age of subjective symptoms compared to the patients with STGD1 (14.91 ± 7.83 vs. 17.59 ± 5.61 years old, *p* = 0.56). However, the age at the first visit of RP patients was significantly later than that of STGD1 patients (44.33 ± 9.39 vs. 26.3 ± 8.75 years, *p* = 0.01). Most of these RP patients demonstrated advanced stage RP with severer macular involvement and poor VA at the most recent visit (LogMAR VA: 1.8 ± 1.04) and extensive lesion areas in the latest FAF examinations (mean ± SD lesion area: 124.61 ± 59.17 mm^2^). There was only one patient (P15) demonstrated relatively macular-sparing phenotype (Supplementary Figure S1). The involved area was positively correlated with the patient’s age (Figure 6C, *p* = 0.32), but was not strongly positively related to VA (Figure 6D, *p* = 0.58).

### 3.5. Cone-Rod Dystrophy

**Figure 7:**
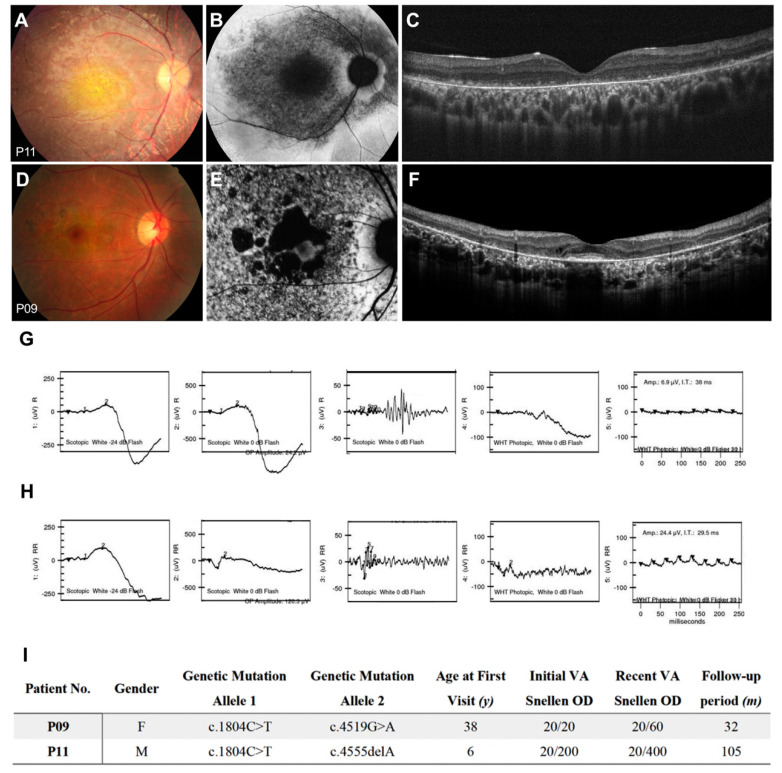
Clinical presentations of *ABCA4*-associated cone-rod dystrophy. (**A**) P11 with macula depigmentation in fundus color, (**B**) DDAF lesions in the central macula, diffuse questionably decreased autofluorescence (QDAF) area extends to arcades, with the peripapillary area spared. (**C**) Diffuse loss of the ellipsoid zone and retinal pigment epithelium. (**D**) P09 with perifoveal depigmentation with a foveal sparing pattern. (**E**) Well demarcated perifoveal DDAF lesions surround the central foveal area with a heterogeneous background. (**F**) Diffuse loss of the ellipsoid zone and retinal pigment epithelium with preserved fovea. (**G**) Electroretinography of P11 showed a decreased amplitude of b waves in a scotopic environment with dim light, decreased b waves in a scotopic environment with bright light, decrease b/a ratio; nearly flat a, b waves in photopic environment, nearly flat waves in photopic flicker (**H**) Electroretinography of P09 with a decreased amplitude of b waves in a scotopic environment with dim light, decreased amplitude of a, b waves in a scotopic environment with bright light; decreased b wave amplitude in a photopic environment, and decreased amplitude in photopic flicker. (**I**) Clinical presentations of two patients with cone-rod dystrophy.

Two patients in our cohort (P09, P11) presented a distinctive phenotype from the others. One had extensive RPE degeneration in nearly the whole macular area in FAF imaging (Figure 7A–C). The other had numerous patchy RPE atrophy around the parafoveal area and diffuse RPE stippling outside (Figure 7D–F). Both patients showed decreased cone function and moderately decreased rod function in electroretinograms (Figure 7G,H) and should be classified as CRD. In these two patients, P09 had a fovea-sparing phenotype and better-preserved central vision. Their clinical presentations are described in detail in Figure 7I.

### 3.6. Genetic Spectrum of ABCA4-Associated Retinal Dystrophies in Taiwanese

**Figure 8:**
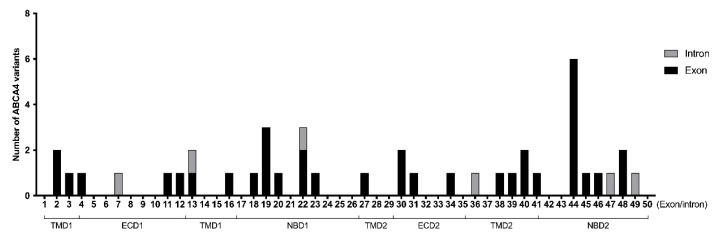
Identified variants within the ABCA4 gene. TMD = transmembrane domain, ECD = extracellular domain, NBD = nucleotide-binding domain.

Panel-based NGS testing was performed for all recruited patients for the identification of disease-causing variants. All patients recruited in this present cohort had two allele disease-causing *ABCA4* variants confirmed according to the American College of Medical Genetics and Genomics guidelines (Supplementary Table S2). A total of 42 different disease-causing variants were found in our patients (Figure 8). Notably, within our cohort, c.1804C>T (p.Arg602Trp) was the most frequent variant, with an extremely high prevalence of 14 patients (14/37, 37.84%) carrying one allele with the c.1804C>T variant. Patients with the c.1804C>T variant may present as any phenotype, and 42.86%, 21.43%, 14.29%, and 21.43% were central type STGD1, dispersion-type STGD1, CRD, and autosomal-recessive RP (arRP), respectively. The relative comparisons between R602W carriers and Non-R602W carriers were shown in Supplementary Table S4. The following frequent variants were c.2894A>G, c.5761G>A, and c.5645T>C, which accounted for 5.41% of the total variants. The most frequent non-point mutation variant was c.101_106del, which accounted for 4.05% of the variants (Supplementary Table S3).

### 3.7. Genotype–Phenotype Correlation

**Figure 9:**
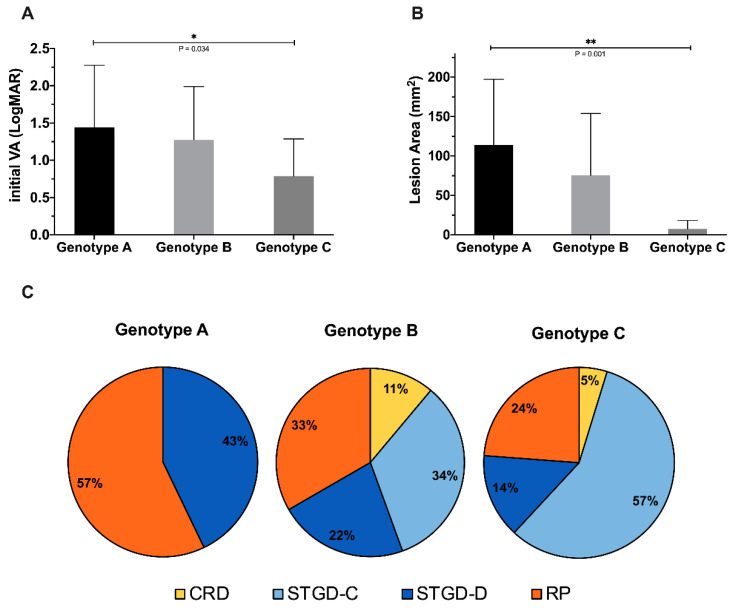
Genotype–phenotype correlations. (**A**) Three genotype groups with corresponding visual acuity at the first visit. (**B**) Three genotype groups with corresponding lesion areas in the first fundus autofluorescence imaging of each patient. (**C**) Distribution of the clinical phenotypes of each genotype group.

To further evaluate the impact of genotype on the clinical presentation, we also classified our patients into three genotype groups in accordance with the ProgStar study [19]. In our cohort, seven, nine, and 21 patients were classified as genotype A (18.92%), genotype B (24.32%), and genotype C (56.76%), respectively. Both the initial VA and lesion area involvement were significantly more severe in group A than in groups B and C (mean ± SD initial LogMAR VA of genotype A: 1.44 ± 0.77, B: 1.14 ± 0.60, C: 0.79 ± 0.23, *p* < 0.01*; mean ± SD initial lesion area of genotype A: 113.79 ± 89.84 mm^2^, B: 75.58 ± 65.42 mm^2^, C: 7.44 ± 6.01 mm^2^, *p* < 0.01*). Genotype A was the most severe group, and genotype C was relatively mild (Figure 9A,B).

In the genotype–phenotype correlation, we further noticed that genotype A tends to lead to arRP (57%) and dispersed type STGD1 (43%). Genotype B included all phenotypes and was associated more with arRP (34%) and central type STGD1 (33%). Genotype C was more likely to present as central type STGD1 (57%; Figure 9C). Genotype A tended to lead to the most extensive type, followed by genotype B and genotype C.

## 4. Discussion

Few studies have been conducted on *ABCA4*-associated retinal dystrophies in Taiwan. Chen et al. reported that *CYP4V2* and *USH2A* were the most common disease-causing genes in Taiwanese IRD patients, and *ABCA4* in China [23,24]. However, most of the patients recruited in that study had a phenotype of RP, which may lead to selection bias in genetic epidemiology. In the present study, we found that *ABCA4*-associated retinal dystrophies are a common disease-causing gene in Taiwan, and their phenotypes could be variable, mainly presenting as STGD1 and arRP, but also some cases of CRD.

For the classification and evaluation of the clinical progression of IRD patients, FAF imaging is useful because it can depict the extent of RPE degeneration. Fujinami et al. classified the FAF of Stargardt disease 1 into three subtypes according to the signal presentation in the fovea and background area [25]. In Fujinami’s study, these three subtypes were not constant and may transit from one type to another as the disease progresses. However, in our cohort, we found that flecks were not frequently present in our patients compared with Caucasian populations described in previous studies [26]. Due to distinctive FAF lesion characteristics from Caucasians, we utilized “central type” and “dispersed type” to classify our STGD1 cases. In our study, no obvious change between groups was noted. A longer period of observation may provide more evidence in the future.

Preservation of VA is always an important issue for patients with IRD. In a previous study, VA was not significantly correlated with the area of DDAF or QDAF, but was significantly associated with involved lesions in the fovea region [27]. In our study, we analyzed the sum of the DDAF and QDAF areas. The lesion areas were significantly associated with VA in the central type group *(p* < 0.01*), but not in the dispersed type group *(p* = 0.245). This may be because the central type lesion was more inclined to involve the fovea and parafoveal areas than the dispersed type.

A foveal sparing phenotype was noted in three of the 37 patients. This phenomenon has been described in previous report [28,29], and was believed to be a milder presentation of mild variants and late-onset STGD1 disease with slower progression [30]. The average onset age of fovea-spared patients in our study was 36.33 years, which is significantly later than the average of the total cohort (36.33 versus 15.80 years, *p* < 0.01). They also had better preservation of their central vision than others (LogMAR VA 0.13 versus 0.75, *p* < 0.01*). Disease-causing *ABCA4* variants of these three patients included c.1804C>T (2/6, 33.33%), c.4519G>A (2/6, 33.33%), c.71G>A (1/6, 16.67%), and c.4070C>A (1/6, 16.67%). Among these variants, c.4519G>A, c.71G>A has been reported in late-onset STGD1 [29,30], but no foveal sparing pattern was reported in patients carrying c.1804C>T and c.4070C>A. To the best of our knowledge, the foveal sparing phenotype has been described in several retinal dystrophies, including STGD1 and age-related macular dystrophy [31,32], but not in patients with CRD. Here, we described a case of late-onset CRD (P09) with a foveal sparing pattern and preserved initial VA, which may provide a new aspect of the phenotype of CRD.

Several studies have attempted to establish the natural history of *ABCA4*-associated retinal degeneration and disease progression [21,22,33]. In our study, similar to the report of Cideciyan, A.V., et al. [34], quicker progression occurred in younger patients. This curve should be of importance to scientists attempting to develop therapeutic interventions for *ABCA4*-associated retinal degeneration, such as gene therapy [35,36] or pharmacological agents [37,38]. Detailed analysis of the natural history of *ABCA4*-associated retinal degeneration may provide an appropriate time window for intervention in further clinical trials.

A total of 42 different disease-causing variants were found in our 37 patients. The most frequent variant, c.1804C>T, which was reported to be a severe missense variant associated with rapid progression, had a significantly high incidence rate (14/37, 37.84%) [39]. *ABCA4* variants are believed to have ethnic and geographic differences. For example, c.3894A>G was found at a high frequency in the Danish population [40] and also represented 5.41% of variants in our study. Variant c.5882G>A was found in half of the patients in South Asian population but was not found in our group [13]. The Taiwanese population is believed to have a shared ancestor from Han China [17]. In the Chinese cohort, variants c.101_106del, c.2894A>G [41], and c.2424C>G [42] were reported as frequent variants with over 5% prevalence in their study, and three of the above variants accounted for 12.11% of the total variants in our study. However, the most frequent variants in our study (c.1804C>T) were not common in the previous Han Chinese cohort, but were more frequent in Caucasians, as well as in the South African population [43,44,45]. Since most of our recruited patients were probands in their family and denied consanguinity with each other, we do not think the high incidence of c.1804C>T was a coincidence or bias. The potential founder effect of c.1804C>T in Taiwanese may be an issue to explore in the future.

In the early 2000s, it was proposed that a combination of alleles with different severities may lead to distinctive phenotypes, but genotype–phenotype prediction remained challenging [8]. CRD and RP were believed to be more severe phenotypes of *ABCA4*-associated retinal dystrophies [46]. A previous cohort showed that the most prevalent phenotype of *ABCA4*-associated retinal dystrophies was STGD1, followed by CRD, and RP [42,44,47]. However, RP accounted for almost one-third of our patients. To further explore the genotype–phenotype correlation, we classified our patients according to their harboring variants and pathogenicity using the same method as the ProgStar study. Interestingly, our results revealed that genotype A with two null/splicing variants tended to develop RP (57%), compatible with the previous conception that RP may be the more severe phenotype in *ABCA4*-associated retinal degeneration [48]. We noticed that the clinical phenotype of RP decreased with decreased numbers of null/splicing variants, while case numbers of STGD1, especially central type STGD1, presented reverse trends. Therefore, we hypothesize that if the phenotypes are on a spectrum in the order of arRP, CRD, dispersed type STGD1, and central type STGD1, genotype A would tend to lead to the most extensive type, followed by genotypes B and C. It is noteworthy that there was a similar trend in the deterioration of the VA and DAF areas among the three genotype groups. The findings were also novel compared to the previous ProgStar study. The differences between these two studies may be due to the different patient recruitment methods used, as we recruited all kinds of phenotypes while ProgStar focused on STGD1 alone.

There are several limitations to this study. First, although detailed history taking was done in each case, it is still challenging to provide the exact age of onset in retrospect recall. Second, we recruited only patients who underwent long-term observations, mostly the proband of each family, rather than all patients with identified *ABCA4*-associated retinal dystrophies. We did that to explore the progression rate and genetic epidemiology among the *ABCA4* disease-causing variants, preventing statistical bias due to including a few big families that have numerous affected members of the same disease-causing variants.

## 5. Conclusions

In conclusion, our report provides the first analysis in a Taiwanese cohort of *ABCA4*-associated retinal dystrophies and their genetic spectrum, genotype–phenotype correlation, visual preservation, and prediction of disease progression. Younger patients with central type and dispersed type STGD1 progressed quicker, while patients with arRP usually had a poor prognosis. We determined the most prevalent disease-causing variants in Taiwanese, including the most frequent variant, c.1804C>T, which is uncommon in China and other East Asian countries. We also found that different types of genotypes would lead to different phenotypes. Patients with a combination of one or more severe/null variants could have more extensive phenotypes, such as arRP, and dispersed type STGD1, rather than central type STGD1. The extensiveness of retinal involvement might be regarded as a spectrum of *ABCA4*-associated retinal dystrophies. These findings could be important not only for the Taiwanese population, but also for clinicians worldwide.

## Figures and Tables

**Table 1:** Demographic data of the *ABCA4*-associated Stargardt disease 1 (STGD1) and retinitis pigmentosa (RP) patients.

	Stargardt Disease 1		Retinitis Pigmentosa	*p*-Value
	Central Type	Dispersed Type		
Patients (*n = 35*)	15	8	12	
Male (*n*)	7 (46.66%)	3 (37.5%)	4 (33.33%)	
Female (*n*)	8 (53.33%)	5 (62.5%)	8 (66.67%)	
Age at first visit, mean ± SD (*years*)	27.07 ± 10.21	24.88 ± 20.57	44.33 ± 9.39	*p* = 0.038
0–19 y (*n*)	7 (46.66%)	5 (62.5%)	1 (8.33%)	
20–29 y (*n*)	3 (20%)	0	1 (8.33%)	
30–39 y (*n*)	1 (6.67%)	0	2 (16.67%)	
40–49 y (*n*)	1 (6.67%)	1 (12.5%)	3 (25%)	
50–59 y (*n*)	2 (13.33%)	1 (12.5%)	4 (33.33%)	
>60 y (*n*)	1 (6.67%)	1 (12.5%)	1 (8.33%)	
Age at symptom onset, mean ± SD (*years*)	18.73 ± 6.05	15.14 ± 15.21	14.91 ± 7.83	*p* = 0.695
0–19 y (*n*)	9 (60%)	6 (75%)	9 (75%)	
20–29 y (*n*)	4 (26.67%)	1 (12.5%)	2 (16.67%)	
30–39 y (*n*)	0	0	0	
>40 y (*n*)	2 (13.33%)	1 (12.5%)	1 (8.33%)	
Visual acuity at first visit, mean ± SD (*LogMAR*)	0.67 ± 0.16	0.77 ± 0.4	1.63 ± 0.46	*p* < 0.001
Visual acuity at recent visit, mean ± SD (*LogMAR*)	0.98 ± 0.28	0.79 ± 0.73	1.8 ± 1.04	*p* = 0.033
Follow-up period (*months*)	64.5 ± 56.55	73.5 ± 73.4	49 ± 37.86	

## Supplementary Materials

The following are available online at https://www.mdpi.com/2073-4425/11/12/1421/s1, Table S1: 212 known genes associated with IRDs, Table S2: Disease-causing variants in this study, Table S3: Total 42 *ABCA4* variants in this study. Table S4: Comparison of R602W carrier with Non-R602W carrier in our cohort, Figure S1: A case of relatively macular-sparing retinitis pigmentosa.
